# Supplementary material for: Genetic Diversity and Population Structure of an Arctic Tertiary Relict Tree Endemic to China (Sassafras tzumu) Revealed by Novel Nuclear Microsatellite (nSSR) Markers
Source: Plants (Basel). 2022 Oct 13;11(20):2706. doi: 10.3390/plants11202706 (PMC9610890; doi:10.3390/plants11202706)
Supplement: Supplementary file 1 [file plants-11-02706-s001.zip › Figure S1.pdf]

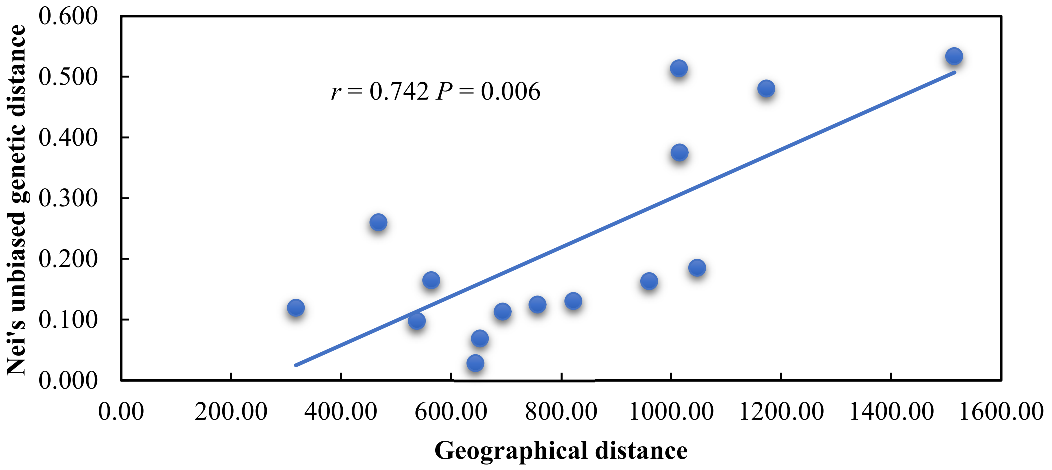

Figure S1. Correlation between Nei's unbiased genetic distance and geographical distance to reflect IBD detected by Mantel test of *S. tzumu*.
